# Wnt2b attenuates HSCs activation and liver fibrosis through negative regulating TLR4 signaling

**DOI:** 10.1038/s41598-017-04374-5

**Published:** 2017-06-21

**Authors:** Yi Yuan, Qiuju Han, Siyu Li, Zhigang Tian, Jian Zhang

**Affiliations:** 1 Institute of Immunopharmaceutical Sciences, School of Pharmaceutical Sciences, Shandong University, Shandong, China; 2 Institute of Immunology, School of Life Sciences, University of Science and Technology of China, Hefei, China

## Abstract

The Wingless-type MMTV integration site family member 2b (Wnt2b) has been found to be a principal mediator of liver development and regeneration. However, the significance of Wnt2b in the pathogenesis of fibrosis-related liver diseases remains undefined. Here, we report that Wnt2b was highly expressed in the fibrotic liver tissues, exhibiting protective effects against activation of hepatic stellate cells (HSCs) and fibrosis progression. We identified a negative regulation of Wnt2b on the toll-like receptor 4 (TLR4) activation-mediated pro-fibrogenic effects. Wnt2b was shown not only to directly suppress LPS-induced HSCs activation, but also to inhibit TLR4-enhanced the sensitivity of HSCs to transforming growth factor beta (TGF-β). Mechanistic study showed that Wnt2b suppresses TLR4 signaling through inhibiting the expression of TLR4 as well as the activation of NF-κB and MAPKs. These findings provided new insights into the pathophysiology of liver fibrosis by characterizing Wnt2b as a novel endogenous suppressor of TLR4 signaling, maintaining tissue homeostasis during the early stage of hepatic fibrosis-associated liver diseases.

## Introduction

Liver fibrosis is clinically associated with the development of cirrhosis and hepatocellular carcinoma (HCC), and has become one of the most significant public health concerns worldwide^1, 2^. Liver fibrosis is a wound-healing response to repeated liver injury and chronic liver inflammation of different etiologies. Fibrogenesis is usually considered as a dynamic process, with the potential to regress by cessation of injury, or to progress into cirrhosis if the key pathways involved in this process were not interrupted successfully at the right time^3^. The activation of hepatic stellate cells (HSCs) is the pivotal event in liver fibrosis. Following the onset of liver injury, the quiescent vitamin A-rich HSCs are activated and differentiate to pro-fibrogenic myofibroblasts (MFBs), which are considered to be the main source of extracellular matrix (ECM) and the major effectors during fibrogenesis^4–6^. Although innovations have been made recent years, the intricate mechanisms underlying HSCs activation in hepatic fibrogenesis are not fully clarified and no anti-fibrotic therapy has yet been approved by FDA^7^.

As an important pattern recognition receptor (PRR), TLR4 has shown to be a primary mediator for HSCs activation and fibrosis progression in the context of chronic liver injury^8, 9^. In experimental hepatic fibrosis models and patients with cirrhosis, the bacterial translocation and lipopolysaccharide (LPS) levels were increased^10^. HSCs highly express TLR4 and are hyperresponsive to LPS^11^. TLR4 promotes HSCs activation either by down-regulating the TGF-β pseudoreceptor Bambi, augmenting TGF-β–mediated HSCs activation^11^, or by negative modulating the functions of miR-146a-5p and miR-29^12, 13^. Moreover, TLR4 augments the accumulation of bone marrow-derived monocytes and Kupffer cells at the damaged sites via enhancing the expression of adhesion molecules and chemokines^14^. Importantly, hyporesponsive TLR4 mutations, such as D299G and T399I single nucleotide polymorphisms (SNPs), have shown to reduce the risk of liver fibrosis in patients with hepatitis C virus infection, promising the pro-fibrogenic role of TLR4 in a clinically relevant setting^15, 16^. Therefore, modulation of the TLR4 signaling might represent a feasible strategy for the treatment or prevention of chronic liver disease.

Wnt2b, also referred to as Wnt13, is a highly conserved secretary glycoprotein of the Wingless-type MMTV integration site (Wnt) family. The Wnt family members regulate multiple biological processes from embryonic development to adult life of all animals^17^, and also show to be implicated in various pathologies such as human malignancies^18^ and metabolic diseases^19, 20^. The functions of Wnt2b in liver physiology have been well demonstrated. Impaired function of Wnt2b in embryos results in a complete absence or severe decrease in hepatic tissue^21^. Additionally, genetic evidence supported a central role of Wnt2b in coordinating early liver development from the multipotent foregut endoderm^17^. The conversion of cholangiocytes to hepatocytes in zebrafish with Wnt2b mutants was impaired following substantial hepatocyte depletion^22^. Shackel *et al*. observed that Wnt2b mRNA increased in human primary biliary cirrhosis (PBC) by using cDNA array analysis^23^. However, the role of Wnt2b in TLR4-associated hepatic inflammation and fibrosis-related liver diseases is still unclear.

In this study, we present the evidence that the level of Wnt2b expression was elevated in the fibrotic liver tissues. Wnt2b displayed an inhibitory effect on HSCs activation and fibrosis progression. Our data demonstrate that Wnt2b may serve as a novel endogenous suppressor of TLR4 signaling-mediated liver fibrosis.

## Results

### Wnt2b expression is elevated in hepatic fibrosis

**Figure 1:**
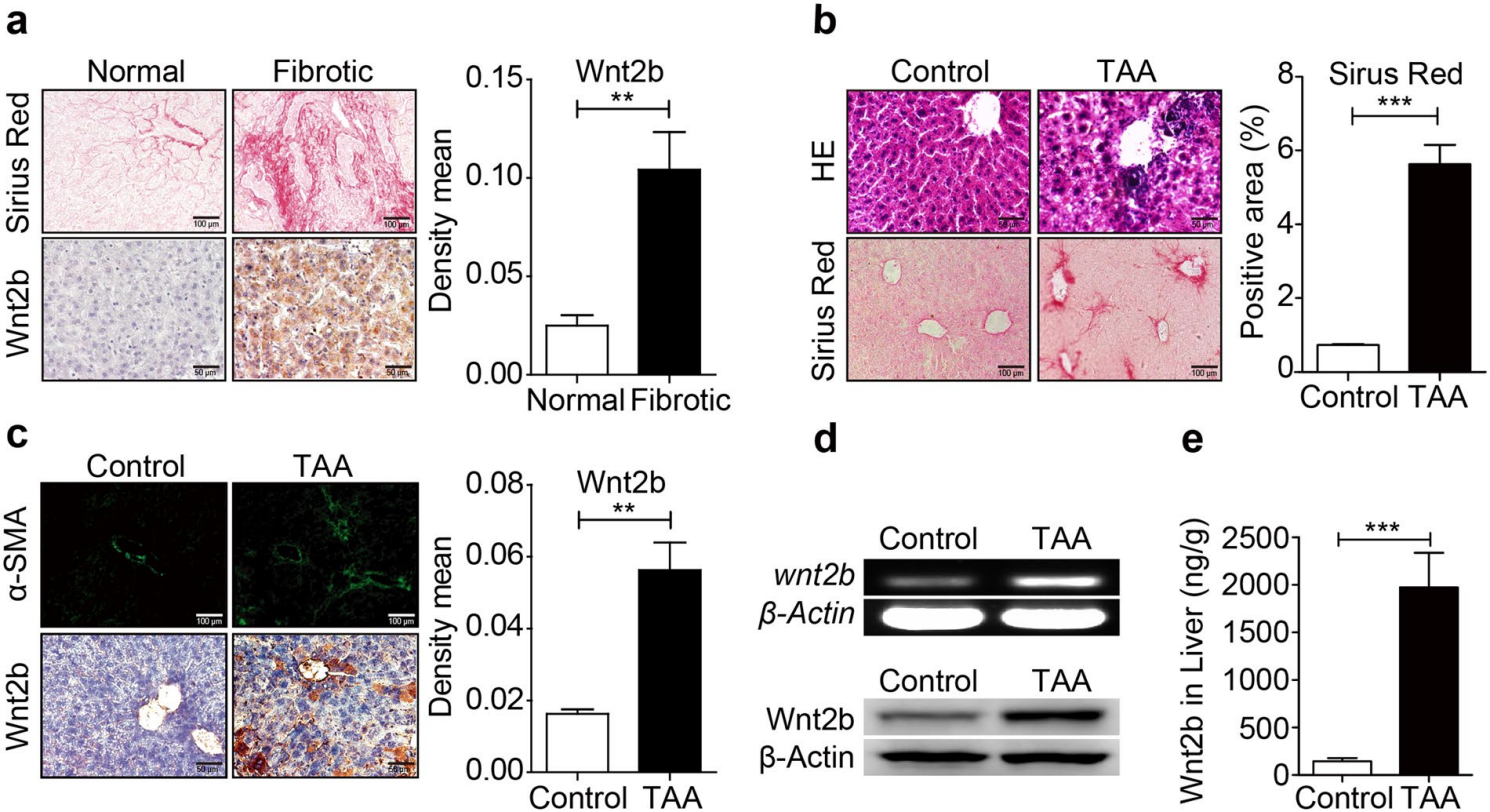
Wnt2b is elevated in hepatic fibrosis. (**a**) Representative Sirius Red staining, IHC staining of Wnt2b in liver tissue microarrays containing healthy controls (n = 9, with a mean age of 43.3 ± 1.4 years) and patients with fibrosis (n = 10, with a mean age of 48.8 ± 2.9 years). Hepatic fibrosis mouse model was induced by TAA, and then the following analyses were performed. (**b**) H&E, Sirius Red staining. (**c**) Immunofluorescence staining of α-SMA and IHC staining of Wnt2b. (**d**) RT-PCR (upper) and Western blotting (lower) of Wnt2b. Cropped blots are displayed; Full-length blots are presented in Supplementary Fig. S5. (**e**) ELISA analysis of Wnt2b in liver homogenate. Statistical analyses provided the mean ± SE (n = 8/group); *P < 0.05, **P < 0.01, ***P < 0.001.

To investigate the potential association of Wnt2b with the development of human fibrotic liver disease, the expressions of Wnt2b were evaluated in Tissue microarrays (TMAs) containing 10 fibrotic subjects (with a mean age of 48.8 ± 2.9 years) and 9 normal controls (with a mean age of 43.3 ± 1.4 years). As shown in Fig. 1a, the fibrotic liver tissues exhibited higher levels of Wnt2b compared to normal controls as demonstrated by immunohistochemistry analysis. Consistently, in liver fibrosis mouse model established by repeated administration of thioacetamide (TAA) (Fig. 1b), hepatic Wnt2b expression was also markedly increased compared to healthy controls, accompanied with highly expressed α-SMA (Fig. 1c). Furthermore, both elevated mRNA (Fig. 1d upper) and protein levels (Fig. 1d lower) of Wnt2b in the fibrotic liver tissues were confirmed by RT-PCR and western blotting, respectively. The amount of Wnt2b in the fibrotic liver homogenate was increased from 143.885 ± 34.63 ng/g to 1972.23 ± 365.10 ng/g (Fig. 1e). Similar results were observed in CCl_4_-treated mice (Supplementary Fig. S1). These findings suggested that Wnt2b might be involved in the pathogenesis of liver fibrosis.

### Wnt2b is mainly produced by hepatocytes during liver fibrogenesis

**Figure 2:**
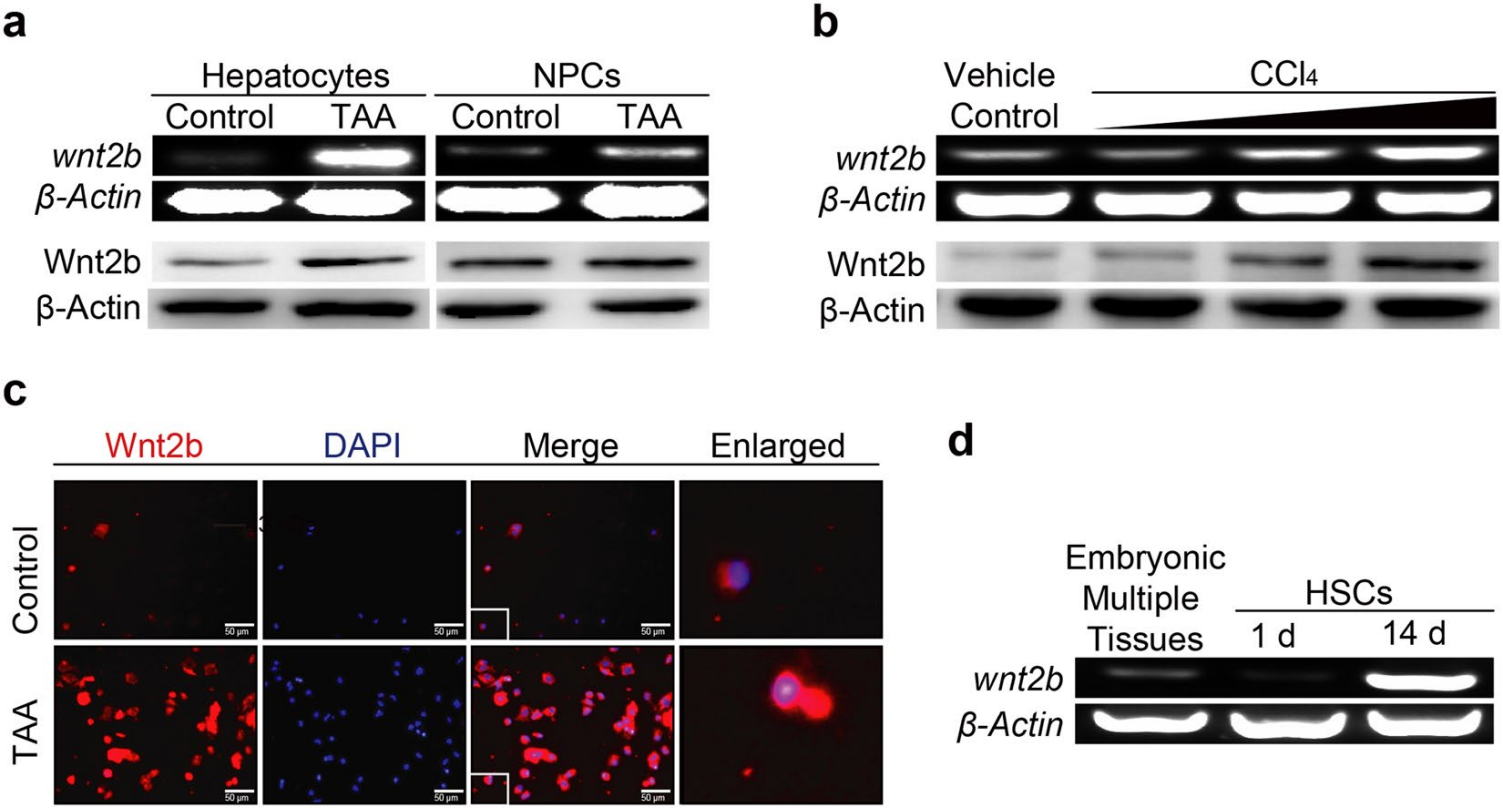
Wnt2b is mainly produced by hepatocytes during liver fibrogenesis. (**a**) mRNA and protein levels of Wnt2b in hepatocytes (left) and NPCs (right) from control and TAA-treated mice, respectively. (**b**) mRNA (upper) and protein (lower) levels of Wnt2b in primary mouse hepatocytes treated with CCl_4_ *in vitro*. (**c**) Immunofluorescence staining of Wnt2b in HSCs from control and TAA-treated mice. (**d**) RT-PCR analysis of Wnt2b in cultured HSCs from naive mice at the indicated points of time. Mouse embryonic tissues served as positive controls. Cropped blots are displayed; Full-length blots are presented in Supplementary Fig. S5. Data are mean ± SEM of three independent experiments; *P < 0.05, **P < 0.01, ***P < 0.001.

To identify the main cellular source of Wnt2b in fibrotic livers, hepatocytes and non-parenchymal cells (NPCs) were isolated from TAA-challenged mice and controls, respectively. As shown in Fig. 2a, the expression of Wnt2b was markedly up-regulated in liver parenchyma obtained from fibrotic mice compared to controls (left), while Wnt2b was slight elevated in NPCs (right). Moreover, when primary hepatocytes isolated from healthy control mice were challenged with hepatotoxic chemicals *in vitro*, both the mRNA and protein levels of Wnt2b were increased in a dose-dependent manner (Fig. 2b), suggesting hepatocytes were the primary cellular source of Wnt2b within fibrotic livers. We further analyzed the changes of Wnt2b in HSCs. As shown in Fig. 2c, the level of Wnt2b in HSCs obtained from fibrotic mice was higher than controls. As quiescent HSCs isolated from healthy control mice were cultured for 14 days *in vitro*, these self-activated HSCs (Supplementary Fig. S2) exhibited higher expression of Wnt2b than the quiescent phenotype (1 d) (Fig. 2d), indicating the levels of Wnt2b in activated HSCs were also increased during liver fibrogenesis.

### Wnt2b attenuates the progression of liver fibrosis

**Figure 3:**
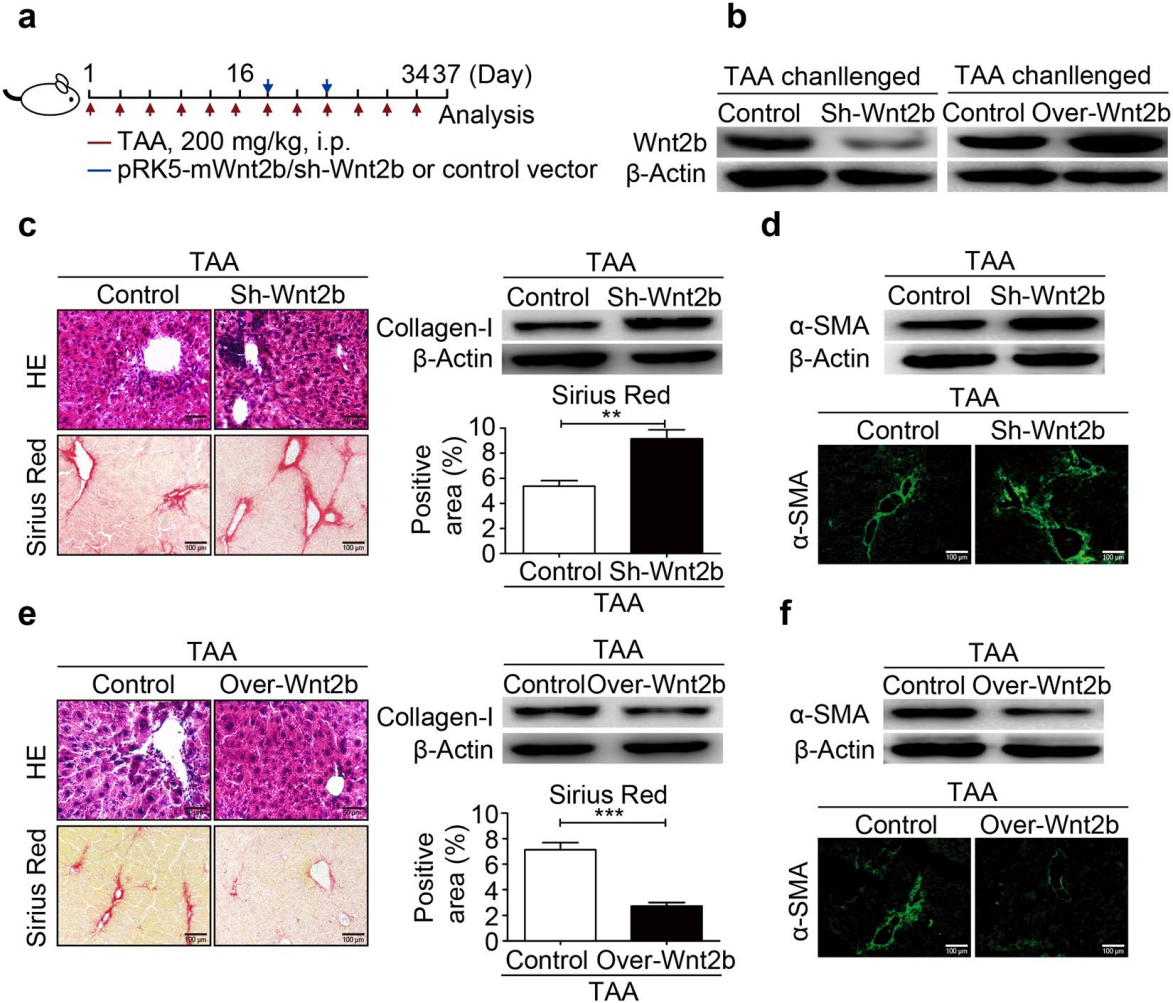
Wnt2b protects against liver fibrosis. (**a**) The schedule for Wnt2b knockdown with shRNA targeting mouse Wnt2b (sh-Wnt2b) or Wnt2b-overexpression with plasmid pRK5-mWnt2b (Over-Wnt2b) under TAA treatment. (**b**) The effects of sh-Wnt2b (left) and pRK5-mWnt2b (right) in TAA-challenged mice livers. (**c**) H&E and Sirius Red staining, Western blotting of Collagen-I, (**d**) Western blotting (upper) and immunofluorescence staining of α-SMA (lower) for liver tissues from mice treated with sh-Wnt2b or control vector. (**e**) H&E and Sirius Red staining, Western blotting of Collagen-I, (**f** ) Western blotting (upper) and immunofluorescence staining of α-SMA (lower) for liver tissues from mice treated with pRK5-mWnt2b construct or control vector. Cropped blots are displayed; Full-length blots are presented in Supplementary Fig. S5. Statistical analysis are mean ± SE (n = 6/group); *P < 0.05, **P < 0.01, ***P < 0.001.

Next, we evaluated whether the level of hepatic Wnt2b expression influenced the degree of liver fibrosis. As shown in Fig. 3a,b, the level of Wnt2b in livers from TAA-challenged mice was knockdown or upregulated by hydrodynamic injection (HD) of shRNA targeting mouse Wnt2b (sh-Wnt2b) and Wnt2b-overexpression plasmid (pRK5-mWnt2b), respectively. Interestingly, sh-Wnt2b markedly promoted total hepatic collagen deposition and CD45 positive cell infiltration, indicating that the decrease in Wnt2b expression accelerated fibrosis progression^24, 25^ (Fig. 3c and Supplementary Fig. S3b). Consistently, an enhanced activation of HSCs was observed in Wnt2b-silencing livers (Fig. 3d), characterized by an increased expression of α-SMA. In contrast to Wnt2b silencing, Wnt2b overexpression resulted in decreased hepatic collagen deposition (Fig. 3e) and CD45 positive cell infiltration (Supplementary Fig. S3c), as well as a marked down-regulation of α-SMA (Fig. 3f). These results collectively suggested that Wnt2b attenuates the progression of liver fibrosis.

### Wnt2b exerts a direct inhibitory effect on HSCs activation

**Figure 4:**
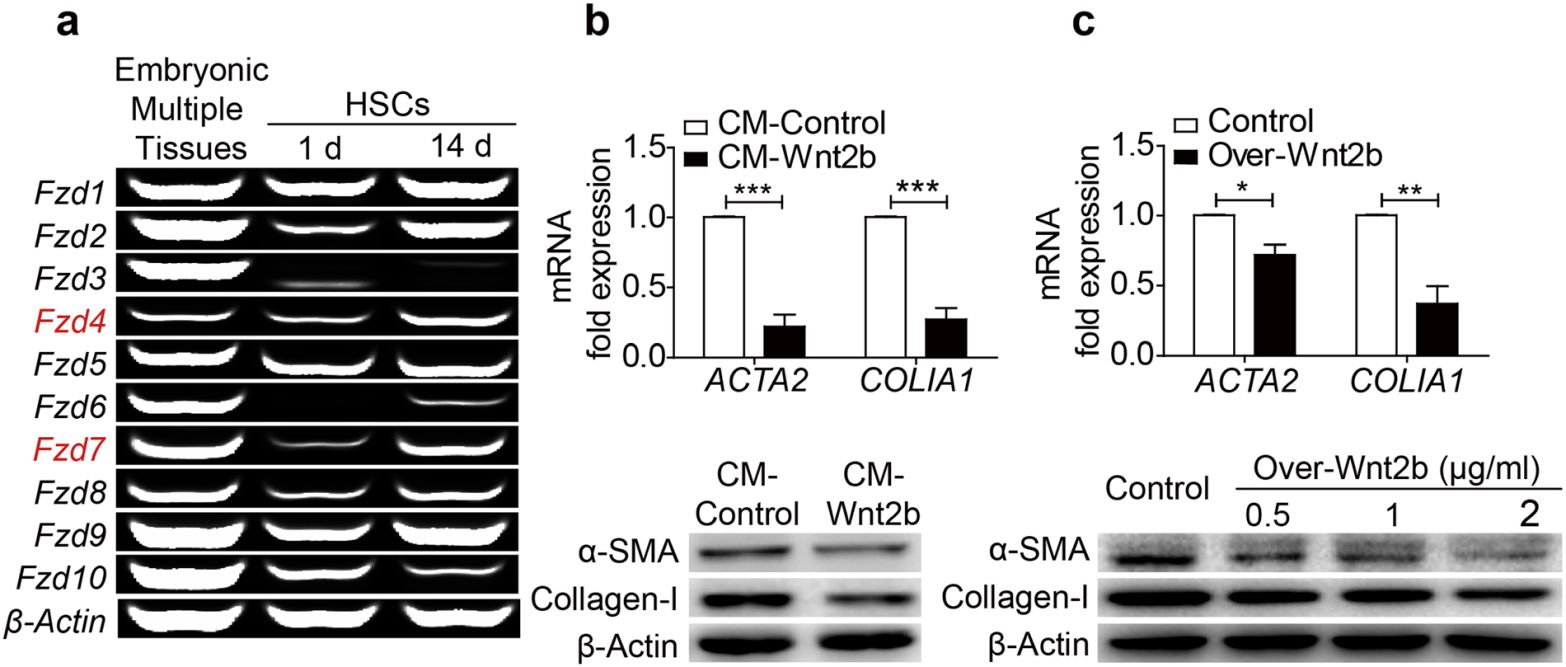
Wnt2b exerts a direct inhibitory effect on HSCs activation. (**a**) RT-PCR analysis of Fzds and LRP5/6 in cultured HSCs from naive mice at the indicated points of time. Mouse embryonic tissues served as positive controls. (**b**,**c**) mRNA and protein levels of α-SMA and Collagen-I in LX2 cells cultured in conditioned medium (CM) collected from HEK293 cells transfected with active Wnt2B-V5 (CM-Wnt2b) or control plasmid (CM-Control) (**b**), as well as in LX2 cells transfected with active Wnt2B-V5 or control plasmid (**c**). Cropped blots are displayed; Full-length blots are presented in Supplementary Fig. S6. Data are mean ± SEM of three independent experiments; *P < 0.05, **P < 0.01, ***P < 0.001.

Since most of the receptors for Wnt signaling, such as Fzd4 and Fzd7^26, 27^, were confirmed to be presented in HSCs, we analyzed whether Wnt signaling is involved in HSCs activation. As shown in Fig. 4a, Fzd4 and Fzd7 receptors were increased in activated HSCs compared to the quiescent phenotype, indicating a direct regulation of Wnt2b signaling on HSCs. To confirm the influence of Wnt2b on HSCs *in vitro*, an immortalized human HSC line LX2 was then used. As shown in Fig. 4b, the expressions of α-SMA and Collagen-Ι were markedly inhibited in LX2 cells cultured with the conditioned medium (CM) from Wnt2b-overexpressing HEK293 cells. Additionally, as LX2 cells transfected with Wnt2b-overexpressing vector, both α-SMA and Collagen-Ι were down-regulated (Fig. 4c). These findings indicated a direct inhibitory effect of Wnt2b on HSCs activation.

### Wnt2b suppresses TLR4 activation-mediated pro-fibrogenic effects

**Figure 5:**
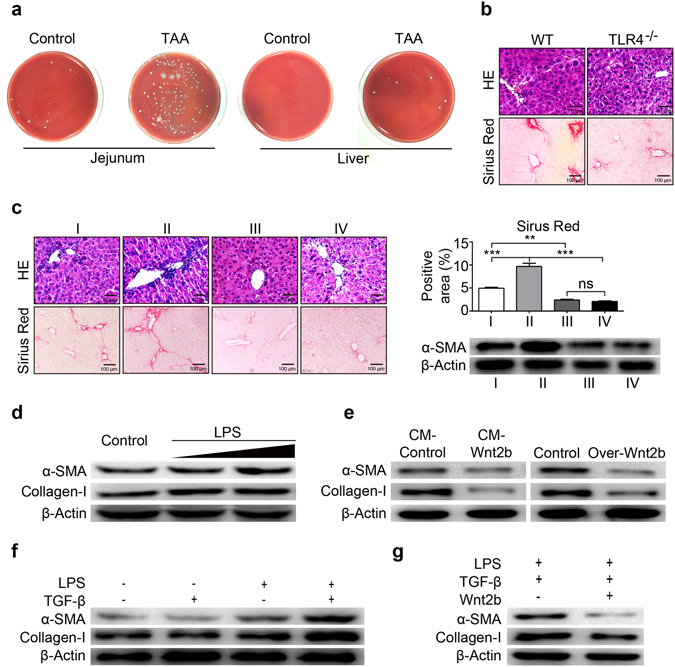
Wnt2b suppresses TLR4 activation-mediated pro-fibrogenic effects. (**a**) Representative images for bacterial growth of jejunum (left) and liver tissues (right) after cultivation on Blood Agar Plates. (**b**) Representative H&E (upper) and Sirius Red staining (lower) of liver tissues from WT mice and *TLR4* ^−/−^ mice after 12 intraperitoneal injections of TAA. (**c**) H&E and Sirius Red staining,Western blotting of α-SMA in liver tissues from mice treated with TAA alone, or combined with sh-Wnt2b construct/TLR4 inhibitor TAK242 (4 mg/kg, i.p.), or all of the three factors given above in combination for 4 weeks. (**d**) Protein levels of α-SMA and Collagen-I in LX2 cells stimulated with LPS (10, 100 ng/ml) for 24 h. (**e**) Effects of Wnt2b on the α-SMA and Collagen-I expressions in LX2 cells stimulated with LPS (100 ng/ml). (**f**) Protein levels of α-SMA and Collagen-I in LX2 cells stimulated with LPS (100 ng/ml) or vehicle for 24 h, and TGF-β (500 pg/ml) or vehicle for an additional 48 h. (**g**) LX2 cells were transfected with active Wnt2B-V5 or control plasmids for 24 h, followed by treatment with LPS ± TGF-β as described in Fig. 5f. The expression of α-SMA and Collagen-I were then detected by Western blot analysis. Cropped blots are displayed; Full-length blots are presented in Supplementary Fig. S7. Statistical analysis provided the mean ± SE (n = 6/group), *P < 0.05, **P < 0.01, ***P < 0.001.

The activation of TLR4 signaling has been demonstrated to be crucial in HSCs activation and fibrosis progression. Consistent with the publications^10, 28^, the small intestinal bacterial overgrowth (SIBO) and bacterial translocation were observed in WT mice fibrotic models (Fig. 5a). Moreover, liver fibrosis was more difficult to be induced in TLR4^−/−^ mice than in WT mice, characterized by lower deposition of collagen (Fig. 5b). Notably, as shown in Fig. 5c, the inhibition of TLR4 pathway with TAK242 abrogated the exacerbated fibrosis effect mediated by silencing Wnt2b. *In vitro* studies, LPS exposure increased α-SMA and Collagen-Ι expressions in LX2 cells in a dose-dependent manner (Fig. 5d), and such pro-fibrogenic effects were markedly attenuated by the presence of Wnt2b (Fig. 5e). In addition, TLR4 signaling was shown to sensitize HSCs to TGF-β stimulation^11^. As shown in Fig. 5f, at the suboptimal dose^29^, TGF-β alone could not induce marked activation of HSCs, while the pre-treatment of LPS enhanced the response of LX2 cells to TGF-β, resulting in strong induction of α-SMA and Collagen-Ι. Importantly, Wnt2b afforded the resistance of LX2 cells to LPS-mediated TGF-β sensitization (Fig. 5g). These results demonstrated a negative regulation of Wnt2b on TLR4-mediated pro-fibrogenic effects, indicating that Wnt2b attenuated HSCs activation and fibrosis progression by suppressing TLR4 pathway.

### Wnt2b disturbs TLR4 signaling transduction

**Figure 6:**
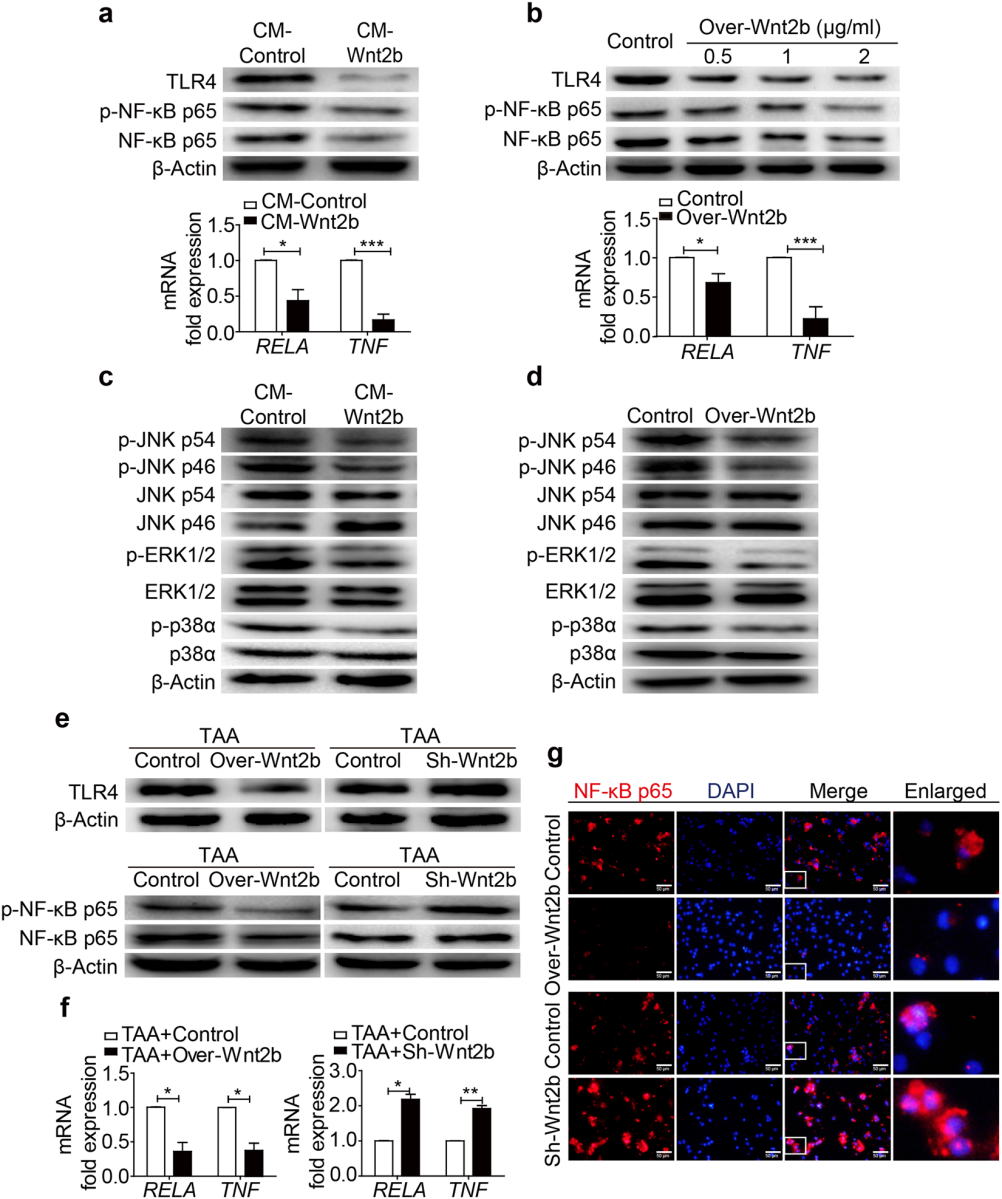
Wnt2b disturbs the TLR4 signaling transduction. (**a**,**b**) Protein levels of TLR4, p-NF-κB p65 (Ser536) and NF-κB p65 (upper), and mRNA levels of RELA and TNF (lower) in LX2 cells cultured in Wnt2b-CM (**a**), as well as in LX2 cells transfected with active Wnt2B-V5 or control plasmids (**b**). (**c**,**d**) Western blotting of the phosphorylation of MAPKs in LX2 cells cultured in Wnt2b-CM (**c)**, and in LX2 cells transfected with active Wnt2B-V5 or control plasmids (**d**). (**e,f** ) Protein levels of TLR4, p-NF-κB p65 (Ser536) and NF-κB p65 (**e**), and mRNA levels of RELA and TNF (**f** ) in liver tissues from mice challenged with TAA combined with HD injection of pRK5-mWnt2b/sh-Wnt2b construct or control vector as depicted in Fig. 3. (**g**) Immunofluorescence staining of NF-κB p65 in HSCs isolated from mice challenged with TAA combined with HD injection of pRK5-mWnt2b (upper) / sh-Wnt2b (lower) construct or control vector as depicted in Fig. 3. Cropped blots are displayed; Full-length blots are presented in Supplementary Fig. S8. Statistical analysis provided the mean ± SE (n = 6/group), *P < 0.05, **P < 0.01, ***P < 0.001.

Subsequently, we wanted to understand the mechanism underlying the inhibitory effect of Wnt2b on TLR4 pathway. We observed the expressions of TLR4 and NF-κB signal transduction were reduced in LX2 cells treated with Wnt2b-CM (Fig. 6a), as well as in LX2 cells transfected with Wnt2b-overexpressing vector (Fig. 6b). Furthermore, the phosphorylation of TLR4 signaling transduction-associated molecules mitogen-activated protein (MAP) kinases^30, 31^, including c-Jun N-terminal protein kinase (JNK), extracellular-regulated kinase (ERK) and P38, was also suppressed in the presence of Wnt2b (Fig. 6c,d). In the parallel *in vivo* experiments, the expressions of TLR4 and TLR4-related signaling intermediates in tissues from fibrotic Wnt2b-overexpressing livers were downregulated compared to WT controls, whereas TLR4 signaling transduction was augmented in Wnt2b-silencing livers (Fig. 6e,f). Meanwhile, Wnt2b overexpression inhibited expression and nuclear translocation of NF-κB in HSCs compared to control, whereas Wnt2b silencing promoted the activation of NF-κB in HSCs (Fig. 6g). These findings collectively suggested that Wnt2b disturbed TLR4 signaling transduction though modulating the expression of TLR4 as well as the activation of TLR4-related signaling intermediates.

## Discussion

**Figure 7:**
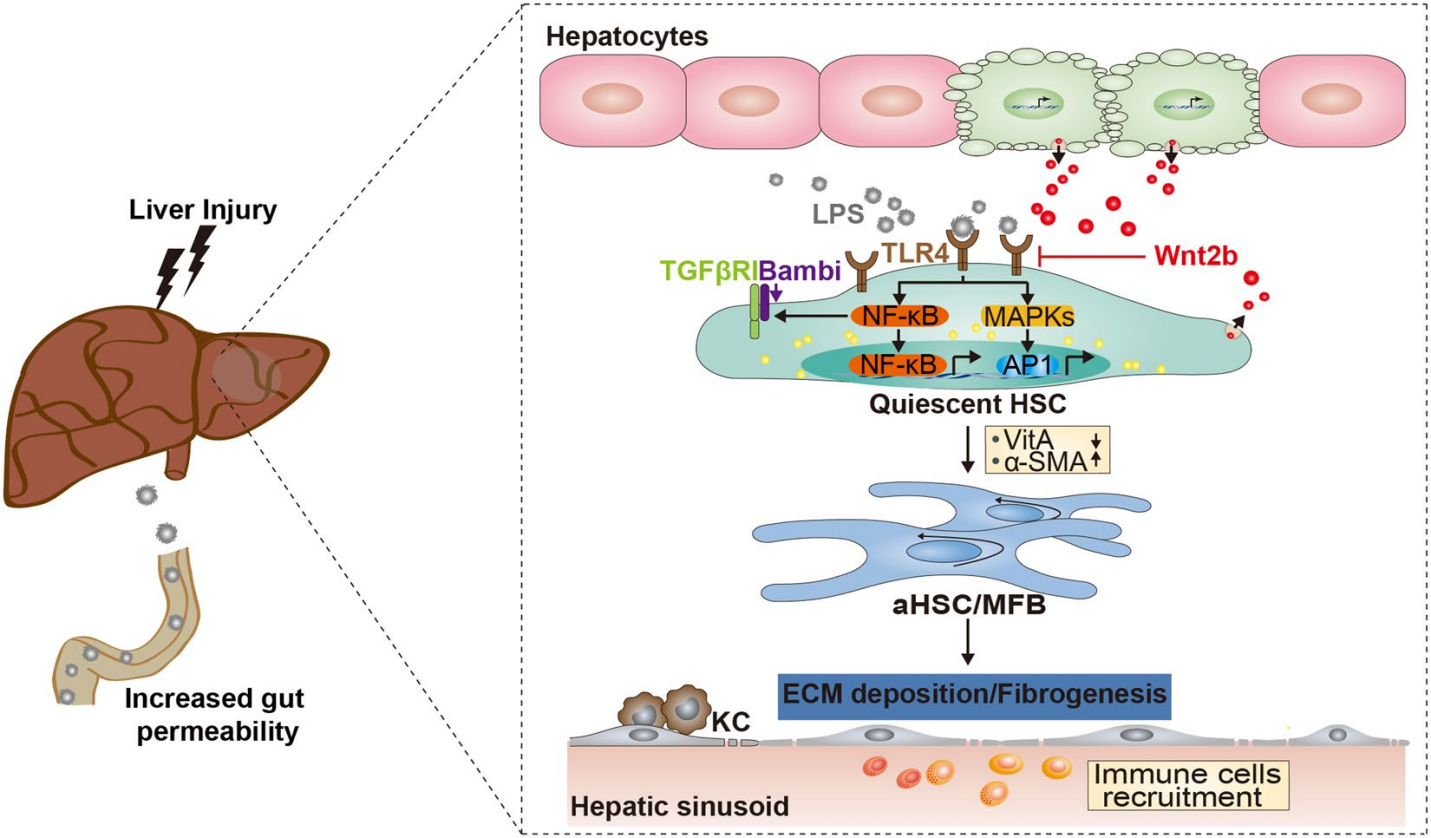
A schematic model for the inhibitory effects of Wnt2b on HSCs activation and liver fibrosis through negative regulating TLR4 signaling.

The activation of TLR4 has a prominent role in driving HSC activation and fibrogenesis^10, 11^. As the first extraintestinal organ to encounter portal venous blood from the gut, liver is prone to exposure to microbial components. Especially, the abnormal quantities of bacterial products could translocate from the gut lumen into the liver because of the increased intestinal permeability under liver injury^14, 28^. Elevations of LPS and TLR4 activation have shown to be not only a feature of end-stage liver disease^32, 33^, but also actively promote HSCs activation and influence disease progression even at the initial stage^34^. Therefore, the investigation for the potential inhibitors of TLR4 represents an attractive strategy for the treatment or prevention of chronic liver disease. The roles of Wnt2b in liver physiology have been thoroughly described, while its significance in hepatic inflammation and fibrosis remains undefined. In the current study, we found Wnt2b was markedly up-regulated in fibrotic livers and acted as an endogenous inhibitor of liver fibrogenesis. Stress-induced Wnt2b exerts a suppressive effect on TLR4 pathway, by which Wnt2b protected against HSCs activation and fibrosis progression. Wnt2b was not only to directly suppress LPS-induced HSCs activation, but also shown to inhibit TLR4-enhanced the sensitivity of HSCs to TGF-β, an important mediator of hepatic fibrosis^35^. Similar to Seki *et al*. reported^11^, we found TLR4 activation could sensitize HSCs to TGF-β stimulation. Pre-treatment of LPS enhanced the response of LX2 cells to a suboptimal dose of TGF-β^29^, while Wnt2b was able to induce the resistance of LX2 cells to LPS-mediated TGF-β sensitization. The mechanism for the inhibitory effect of Wnt2b on TLR4-mediated pro-fibrogenic effects might relate to the following findings that Wnt2b suppressed the expression of TLR4 and the activation of TLR4 downstream signal transduction, including NF-κB and MAPKs. TLR4 induced the enhancement of HSCs activation to TGF-β in an NF-κB-dependent manner^11^. Accordingly, inhibition of NF-κB by Wnt2b could largely cancel the sensitivity of HSCs to TGF-β. In addition, by analyzing the 5′-regulatory region of TLR4 gene, we identified the putative binding sites for the transcription factor activator protein-1 (AP-1) and NF-κB at the upstream of the transcription start site in TLR4 promoter. Since MAPKs could be activated by LPS stimulation and in turn promote the transcription of AP-1 and NF-κB^36, 37^, a TLR4-MAPKs-AP-1/NF-κB positive feedback loop might exist and be involved in HSCs activation. Here, Wnt2b was shown to inhibit the activation of MAPKs in HSCs, raising the possibility that Wnt2b broke TLR4-boosted transcriptional positive feedback. However, the details of the mechanism underlying the inhibitory effect of Wnt2b on TLR4 pathway need to be determined through more exact experiments. And, the possible correlation between Wnt2b and TLR4 in the progression of fibrosis-related liver diseases will be continued in our future work. In addition to HSCs, hepatocytes also expressed most of the receptors for Wnt signaling (data not shown). Although serum ALT levels were not differentially changed by Wnt2b-overexpressing/silencing **(**Supplementary Fig. S4), suggesting that the exacerbated fibrosis effect mediated by Wnt2b silencing might not be directly associated with hepatocytes injury^38^, further investigation on the roles of hepatocytes on liver fibrosis is still important in the future^39^. Nonetheless, the current study expanded our understanding of the complex relationship between the Wnt pathway and the development of fibrosis. Significantly, Wnt2b was identified as a novel endogenous TLR4 suppressor, which displayed suppressive effects on HSCs activation and fibrosis progression (Fig. 7). These findings provided a potential therapeutic candidate which could be suitable for restoring tissue homeostasis during the early stage of liver disease, and indicated a possible role for Wnt2b in sterile inflammation and inflammation-related oncogenesis.

## Materials and Methods

### General methodology

General methodology such as H&E staining, Immunohistochemistry analysis, Sirius red staining, and Isolation of primary HSCs were described in the supplementary materials. The information of the patients and control subjects was available in the Supplementary Table 1. The source of Abs used was listed in Supplementary Table 2. The RT-PCR and qPCR primers used were shown in Supplementary Tables 3 and 4, respectively.

### Ethics Statement

All animal study proposal and protocol were approved by Ethical Committee of Shandong University (License No: LL-201602065). Animal care provided was conformed to the principles in “Guide for the Care and Use of Laboratory Animals” (NIH, 1996). All animal experimental protocols were performed in accordance with “Regulations for the Administration of Affairs Concerning Experimental Animals” (Approved by State Science and Technology Commission, People’s Republic of China, 10/31/1988). For the studies on human tissue samples, the Tissue microarrays (TMAs) were constructed by Shanghai Biochip Co., Ltd. and AlenaBio Co., Ltd., approved by Ethical Committee of Zhejiang province Taizhou Hospital and Henan province Tongxu People’s Hospital. The methods were carried out in accordance with the approved guidelines. All written informed consents were obtained from all subjects in this study.

### Animals and Models of Fibrosis

Pathogen-free male C57BL/6 mice (4–6 weeks) were obtained from HuaFuKang Biological Technology Co., Ltd. (Beijing, China). C57BL/6-derived *TLR4-knockout* mice (*TLR4* ^−/−^, male, 4–6 weeks)^40^ were kindly provided by S.B. Sun (Sun Yat-Sen University, Guangdong, China). Chronic hepatocellular stress-dependent liver fibrosis was induced by 12 intraperitoneal injections of thioacetamide (TAA) (T104039, Aladdin, Shanghai, China) at 200 mg/kg or 8 such injections of carbon tetrachloride (CCl_4_) (Fuyu, Tianjin, China) at 0.8 ml/kg^41^.

### Cell Line and Reagents

LX-2, an immortalized human HSC line with a stable phenotype and biochemical characteristics of activated HSCs^42^, was verified by checking the ICLAC and NCBI databases and used for studies *in vitro*. LPS was purchased from Sigma-Aldrich (L2630, St. Louis, MO, U.S.). Recombinant human TGF-β1 was purchased from PeproTech (100–21C, Rocky Hill, U.S.). TLR4 inhibitor (TAK-242) was purchased from Calbiochem (614316, Darmstadt, Germany). NF-κB inhibitor (BAY11–7082) was purchased from Beyotime Biotechnology (S1523, Shanghai, China).

### Mouse Plasmids and Hydrodynamic Injection

pRK5-mWnt2b encoding murine Wnt2b was provided by Chris Garcia and Jeremy Nathans (Addgene plasmid # 42275)^43^. The shRNA plasmid targeting mouse Wnt2b was purchased from OriGene (Gene ID 22414, Beijing, China). Plasmids were diluted in 2 ml saline (0.9% NaCl) for each mouse, and then hydrodynamically injected through the caudal vein within 8 to 10 s. The efficacy of plasmids delivering into the livers was examined by Western blot.

### Human Plasmids and Cell Transfection

Active Wnt2B-V5 encoding homonine Wnt2b was a gift from Xi He (Addgene plasmid # 43808)^44^. LX-2 cells were plated at 5 × 10^5^ cells/well in 6-well plates, and the transfection was performed with Lipofectamine 2000 (11668–019, Invitrogen^TM^, CA, U.S.) in accordance with the manufacturer’s instructions. Cells were analyzed 24 h after transfection unless stated otherwise.

### Statistical analysis

Data were presented as the mean ± standard error of the mean (SEM). All the *in vitro* experiments were performed at least three times independently. *In vivo* studies, mice were randomly assigned to each group and the minimum number of mice used for each assay was three. Statistical analysis between two groups was performed using Student’s t-test and analysis between multiple groups was analyzed by one-way ANOVA with Bonferroni correction. Differences were considered statistically significant at *P* value < 0.05. (*P value < 0.05; **P value < 0.01; ***P value < 0.001).

### Data Availability

All data generated or analyzed during this study are included in this published article (and its Supplementary Information files).

## Electronic supplementary material

Supplementary Materials
