# Supplementary material for: Wnt2b attenuates HSCs activation and liver fibrosis through negative regulating TLR4 signaling
Source: Sci Rep. 2017 Jun 21;7:3952. doi: 10.1038/s41598-017-04374-5 (PMC5479809; doi:10.1038/s41598-017-04374-5)
Supplement: Supplementary file 1 — Supplementary Materials [file 41598_2017_4374_MOESM1_ESM.doc]

**Supplementary data to:**

**Wnt2b attenuates HSCs activation and liver fibrosis through negative regulating TLR4 signaling**

Yi Yuan, Qiuju Han, Siyu Li, Zhigang Tian, Jian Zhang

**Table of Contents**

**Supplementary Materials and Methods**.………………………………………..…..2

**Supplementary References**…………………………………………………..…..…..4

**Supplementary Table 1**………………………………………………………………5

**Supplementary Table 2**………………………………………………………………6

**Supplementary Table 3**………………………………………………………………7

**Supplementary Table 4**………………………………………………………………8

**Supplementary Figure 1**…………………………………………………………… 9

**Supplementary Figure 2**…………………………………………………………....10

**Supplementary Figure 3**……………………………………………….…....……...11

**Supplementary Figure 4**……………………………………………….…....……...12

**Supplementary Figure 5**……………………………………………….…....……...13
 **Supplementary Figure 6**……………………………………………….…....……...14 **Supplementary Figure 7**……………………………………………….…....……...15

**Supplementary Figure 8**……………………………………………….…....……...16


**Supplementary Materials and Methods
Histology and Immunohistochemistry**Mice liver tissues were fixed with 4% paraformalehyde for 24 h and embedded in paraffin. 5 µm-thick sections were prepared for use. Tissue microarrays (TMAs), containing 10 fibrotic subjects (with a mean age of 48.8 ± 2.9 years) and 9 normal controls (with a mean age of 43.3 ± 1.4 years) were constructed by Shanghai Biochip Co., Ltd. (HLiv-HCC060CD-01) and AlenaBio Co., Ltd. (LVN241). These subjects were age matched (P=0.1215). Detailed information of the patients and control subjects was available in the **Supplementary Table 1**.The extent of fibrosis was assessed by Sirius red staining using a standard protocol. Briefly, sections were stained with 0.4% Sirius red in saturated picric acid for 0.5 h. At least five different fields on each slide were measured. The positively stained area was selected by threshold adjustment on a gray scale picture using Image J software (NIH, U.S.) and the ratio of positively stained area/total area was then calculated. For immunohistochemical analysis, protein expression was visualized by 3,3’-diaminobenzidine tetrahydrochloride (DAB) staining. At least four random fields were examined for each sample. The stained area and Integrated Optical Density (IOD) were measured using Image-pro Plus 6.0 software, and the density mean, equal to (IOD SUM)/area, was then calculated.**Immunofluorescence**Cultured primary HSCs Cells were fixed in 4% paraformaldehyde for 15 min at room temperature and washed with PBS-T (PBS containing 0.1% Tween-20) thrice. Then cells were blocked with 5% Goat Serum (C0265, Beyotime, Shanghai, China) for 30 min and incubated with anti-bodies of interest at 4°C overnight. After washing with PBS-T, cells were incubated with a fluorochrome-conjugated secondary antibody (A23320, Abbkine, CA, U.S.; 1:200) for 1 h. Diaminophenylindole (DAPI) was used for nucleus staining.

Mouse liver sections 5 µm-thick frozen liver sections were fixed in 10% buffered formalin for 10 min, and blocked with 5% Goat Serum (C0265, Beyotime, Shanghai, China) for 30 min, followed by incubation with mouse anti-α−SMA antibody (Ab7817, Abcam, Cambridge, U.K.; 1:200) at 4°C overnight. After washing with PBS-T, sections were incubated with fluorochrome-conjugated secondary antibodies (A23210, Abbkine, CA, U.S.; 1:200) for 1 h. Diaminophenylindole (DAPI) was used for nucleus staining.  **Isolation and Culture of HSCs**
Primary HSCs were isolated using an established method^1^. Briefly, mouse livers were perfused *in situ* with collagenase type I (1723329, Gibco, CA, U.S.), followed by OptiPrep (AS1114542, Axis-Shield, Oslo, Norway) density gradient centrifugation. Cell viability was determined by trypan blue exclusion. Cell purity was confirmed by examining three major characteristics of HSCs, its star-like shape, perinuclear lipid droplets, and vitamin A-specific auto-fluorescence^2^.
**Isolation of NPCs** Mice hepatic non-parenchymal cells (NPCs) were collected as previously described^3^. Briefly, mouse livers were perfused *in situ* with collagenase type I (1723329, Gibico, CA, U.S.). Hepatocytes were excluded with low speed (400 rpm) centrifugation followed by high-speed (2,000 rpm) centrifugation to obtain the NPCs, which were then further enriched over a 40% Optiprep (AS1114542, Axis-Shield, Oslo, Norway) gradient.
**Bacterial Overgrowth and Bacterial Translocation Analysis** The intestinal bacterial overgrowth and bacterial translocation were assessed by conventional culture techniques^4^, intestinal segment and liver tissues were harvested in a sterile fashion and weighed before homogenization, respectively, then lysed with 1% Triton X-100 in PBS. Lysates were plated onto Blood Agar Plates under sterile conditions and incubated for 72 h at 37^o^C followed by the counting of colony-forming units (CFUs). **Western blot analysis**
Thirty micrograms of proteins extracted from liver fragments or cultured cells were separated by sodium dodecyl sulfate-polyacrylamide gel electrophoresis (SDS-PAGE) and then transferred to polyvinylidine fluoride membranes (IPVH00010, Merck Millipore, Merck’s life science business, Darmstadt, Germany) according to standard procedures. After blocked with 5% non-fat milk in TBS-T, membranes were incubated with primary antibodies (Abs) of interests (listed in **Supplementary Table** **2**) at a dilution of 1:500-1000. Then proteins were visualized using Immobilon Western Chemiluminescent HRP Substrate (WBKLS0500, Merck Millipore, life science business of Merck KGaA, Darmstadt, Germany) and detected with Alpha Ease FC software (Bio-Rad, Hercules, CA, U.S.). *In vivo* studies, Western blot bands were pooled samples from at least 3 mice

**Reverse transcriptase polymerase chain reaction (RT-PCR)**
Total RNA was extracted from liver tissues or cultured cells using the Trizol Reagent (15596018, Ambion®, Invitrogen™, CA, U.S.) according to the manufacturer’s recommendations. The first stranded cDNA was synthesized using 2 µg total RNA with FastQuant RT Kit (KR106-02, TIANGEN, Beijing, China). Standard PCRs were performed in a total reaction volume of 25 μl as previously described. The PCR products were then electrophoresed in 1.5-2% agarose gels containing 5 µg/ml Nucleic acid dye and quantitatively analyzed with AlphaEaseFC software (Bio-Rad, Hercules, CA, U.S.). The primers used were listed in **Supplementary Table** **3**. *In vivo* studies, RT-PCR bands were pooled samples from at least 3 mice

**Quantitative Real-time PCR (****qPCR)**
Gene expression was measured by real-time PCR using FastStart Universal SYBR Green Master (Rox) (04913914001, Roche, Basel, Switzerland). Samples were run in triplicates, expression values were normalized against reference genes (β-actin, or GADPH), and fold-changes were calculated with respect to the control group using the 2^-∆∆^Ct. The primers used were shown in **Supplementary Table** **4**.

**Hepatic Immune Cell Isolation and Flow Cytometry Analysis**

The extent of immune cell infiltration was quantified by analyzing the number of CD45 positive cells in fibrotic livers from *in vivo* models^5, 6^. Briefly, mouse livers were freshly harvested, and mononuclear cells (MNC) were separated by density gradient centrifugation. Then, cells were counted by an automated cell counter (TC20, Bio-Rad, CA, U.S.) and incubated with the indicated fluorescent Abs (PE-Cy5.5-conjugated IgG isotype, PE-Cy5.5-conjugated anti-CD45 mAb, eBioscience^TM^, Thermo Fisher Scientific, CA, U.S.). The stained cells were measured with flow cytometer (FACSCalibur, BD Biosciences, CA, U.S.), and the data were analyzed with FCS Express V3 (De Novo^TM^ Software, CA, U.S.).

**Serum biochemistry and Enzyme linked immunosorbent assay (ELISA)**
Serum levels of alanine aminotransferase (ALT) were measured using standard enzymatic procedures according to the manufactures’ instruction (C009-2, JianCheng Bioengineering Institute, NanJing, China). Wnt2b concentrations in the liver homogenate were measured using the Mouse Wnt2b Elisa Kit (M30451-09, Boyun Biotech. Co., Ltd, Shanghai, China) in accordance with the manufacturer’s instructions.

**Supplementary references**
[1] Chang, J. *et al*. Activation of Slit2-Robo1 signaling promotes liver fibrosis. *Journal of hepatology* **63,** 1413-1420; DOI: 10.1016/j.jhep.2015.07.033 (2015).
[2] Maschmeyer, P., Flach, M., Winau, F. Seven steps to stellate cells. *Journal of visualized experiments* **51**, 2710; DOI: 10.3791/2710 (2011).

[3] Connolly, M. K. *et al.* In liver fibrosis, dendritic cells govern hepatic inflammation in mice via TNF-alpha. *The Journal of clinical investigation* **119**, 3213-3225; DOI: 10.1172/JCI37581 (2009).

[4] [Fouts, D. E](https://www.ncbi.nlm.nih.gov/pubmed/?term=Fouts DE%5BAuthor%5D&cauthor=true&cauthor_uid=22326468)., [Torralba, M](https://www.ncbi.nlm.nih.gov/pubmed/?term=Torralba M%5BAuthor%5D&cauthor=true&cauthor_uid=22326468)., [Nelson, K. E](https://www.ncbi.nlm.nih.gov/pubmed/?term=Nelson KE%5BAuthor%5D&cauthor=true&cauthor_uid=22326468)., Brenner, D. A., [Schnabl, B](https://www.ncbi.nlm.nih.gov/pubmed/?term=Schnabl B%5BAuthor%5D&cauthor=true&cauthor_uid=22326468). Bacterial translocation and changes in the intestinal microbiome in mouse models of liver disease. *Journal of hepatology* **56**, 1283-1292; DOI: 10.1016/j.jhep.2012.01.019 (2012).

[5] Heinrichs, D. *et al*. The chemokine CCL3 promotes experimental liver fibrosis in mice. *PLoS One* **8**, 66106; DOI: 10.1371/journal (2013).

[6] Vasseur, P. *et al*. High-Fat Diet-Induced IL-17A Exacerbates Psoriasiform Dermatitis in a Mouse Model of Steatohepatitis. *The American Journal of Pathology* **186**, 2292-2301; DOI: 10.1016/j.ajpath.2016.05.012 (2016).

**Supplementary Table 1. The information of the patients and control subjects in TMAs study**

| **No.** | **Histological type** | **Pathological type** | **Age** | **Sex** |
| --- | --- | --- | --- | --- |
| 1 | Normal liver | Normal liver | 40 | M |
| 2 | Normal liver | Normal liver | 40 | M |
| 3 | Normal liver | Normal liver | 40 | M |
| 4 | Normal liver | Normal liver | 40 | M |
| 5 | Normal liver | Normal liver | 42 | M |
| 6 | Normal liver | Normal liver | 43 | M |
| 7 | Normal liver | Normal liver | 45 | M |
| 8 | Normal liver | Normal liver | 50 | M |
| 9 | Normal liver | Normal liver | 50 | F |
| 10 | Cirrhosis | Cirrhosis | 38 | M |
| 11 | Cirrhosis | Cirrhosis | 43 | M |
| 12 | Cirrhosis | Cirrhosis | 43 | M |
| 13 | Cirrhosis | Cirrhosis | 49 | M |
| 14 | Cirrhosis | Cirrhosis, with liver cell denaturation | 53 | M |
| 15 | Cirrhosis & Hyperplasia | Nodular cirrhosis | 57 | M |
| 16 | Cirrhosis & Hyperplasia | Nodular cirrhosis, with foci of atypical hyperplasia | 60 | M |
| 17 | Pericarcinous tissues | Cirrhosis | 39 | M |
| 18 | Pericarcinous tissues | Nodular cirrhosis | 42 | M |
| 19 | Pericarcinous tissues | Cirrhosis with chronic inflammation | 64 | F |

**Supplementary Table 2. Anti-bodies used in this study**

| **Antibodies** | **Source** | **MW (kDa)** | **Product code & Supplier** |
| --- | --- | --- | --- |
| α-SMA | Rabbit | 43 | ab5694, Abcam, Cambridge, U.K. |
| α-SMA | Mouse | 37 | ab7817, Abcam, Cambridge, U.K. |
| Wnt2b | Rabbit | 44 | ab178418, Abcam, Cambridge, U.K. |
| p-NF-κB p65 (Ser536) | Rabbit | 65 | #3033, Cell Signaling Technology, Massachusetts, U.S. |
| NF-κB p65 | Rabbit | 65 | #8242, Cell Signaling Technology, Massachusetts, U.S. |
| CyclinD1 | Rabbit | 36 | #2922, Cell Signaling Technology, Massachusetts, U.S. |
| Erk1/2 | Rabbit | 42, 44 | #4695, Cell Signaling Technology, Massachusetts, U.S. |
| p-Erk1/2  (Thr202/Tyr204) | Rabbit | 42, 44 | #14227S, Cell Signaling Technology, Massachusetts, U.S. |
| Histone H2A.X | Rabbit | 15 | #7631, Cell Signaling Technology, Massachusetts, U.S |
| TLR4 | Rabbit | 95 | sc-10741, Santa Cruz Biotechnology, CA, U.S. |
| p38α | Mouse | 38 | sc-535, Santa Cruz Biotechnology, CA, U.S. |
| p-p38α  (Thr180/Tyr182) | Rabbit | 38 | sc-17852-R, Santa Cruz Biotechnology, CA, U.S. |
| JNK | Mouse | 46, 54 | sc-7345, Santa Cruz Biotechnology, CA, U.S. |
| p-JNK  (Thr183/Tyr185) | Mouse | 46, 54 | sc-6254, Santa Cruz Biotechnology, CA, U.S. |
| β-Actin | Mouse | 43 | sc-8432, Santa Cruz Biotechnology, CA, U.S. |
| Collagen-I | Rabbit | 130 | WL0088, Wanleibio, Beijing, China |

**Supplementary Table 3. Sequence (5’ – 3’) of primers used for RT-PCR in this study**

| **Species** | **Gene (Symbol)** | **Forward Primer** | **Reverse Primer** |
| --- | --- | --- | --- |
| Mouse | β-actin (ACTB) | GGACTCCTATGTGGGTGGCGAGG | GGGAGAGCATGCCCTCGTAGAT |
|  | Wnt2b (Wnt2b) | CGTTCGTCTATGCTATCTCGTCAG | ACACCGTAATGGATGTTGTCACTAC |
|  | Fzd1 (Fzd1) | AACTTTGTGCCGAAGCACTC | GGTCTGGTTGTACGCGATGT |
|  | Fzd2 (Fzd2) | TCGCCTACAACCAGACCATC | CATTGGAAGCCGAACTTGT |
|  | Fzd3 (Fzd3) | GGGTTGGAAGCAAAAAGACA | CTCCCTGCTTTGCTTCTTTG |
|  | Fzd4 (Fzd4) | CAACCTGTGTGATTGCCTGT | TGTGTGTGGGCTGAAGTGTT |
|  | Fzd5 (Fzd5) | GGCATCTTCACCCTGCTCTA | TTCCTCTCCAAGCCACTCTG |
|  | Fzd6 (Fzd6) | GGCTGAAGGTCATTTCCAAG | TGAACAGGCAGAGATGTGGA |
|  | Fzd7 (Fzd7) | GAAGCTGGAGAAGCTGATGG | ATCTCTCGCCCCAAATCTCT |
|  | Fzd8 (Fzd8) | CGGTGGTCTTTCTCCTTGTC | TAGAAAAGGCAGGCGACAAC |
|  | Fzd9 (Fzd9) | AGAGCCTGTGCTACCGAAAA | CCCCCTGTGTCTCACTTGTC |
|  | Fzd10 (Fzd10) | GACACCTGACTGCCTGATGA | ACAACCAGCCAACCAAGAAA |

**Supplementary Table 4. Sequence (5’ – 3’) of primers used for qPCR in this study**

| **Species** | **Gene (Symbol)** | **Forward Primer** | **Reverse Primer** |
| --- | --- | --- | --- |
| Human | β-actin (ACTB) | CACTGTGTTGGCGTACAGGT | TCATCACCATTGGCAATGAG |
|  | GADPH | GAAGGTGAAGGTCGGAGT | CATGGGTGGAATCATATTGGAA |
|  | α-SMA (ACTA2) | GATCTCAGTGCAGAGGCTCG | TTTGCTTGTCCAGGTGGTCC |
|  | Collagen-I (COL1A1) | GGGACACAGAGGTTTCAGTGGT | GCACCATCATTTCCACGAGC |
|  | NF-κB p65 (RELA) | ATGTGGAGATCATTGAGCAGC | CCTGGTCCTGTGTAGCCATT |
|  | TNF-α (TNF) | GCATGATCCGGGACGTGGAG | CCCTGGGGAACTCTTCCCTCT |
|  | Cyclin D1(CCND1) | CACGGACTACAGGGGAGTTTTGT | ACTCTGCTGCTCGCTGCTAC |
| Mouse | β-actin (ACTB) | AGAGGGAAATCGTGCGTGAC | CAATAGTGATGACCTGGCCGT |
|  | NF-κB p65 (RELA) | TGCGATTCCGCTATAAATGCG | ACAAGTTCATGTGGATGAGGC |
|  | TNF-α (TNF) | GATCGGTCCCCAAAGGGATG | CCACTTGGTGGTTTG TGAGTG |

**
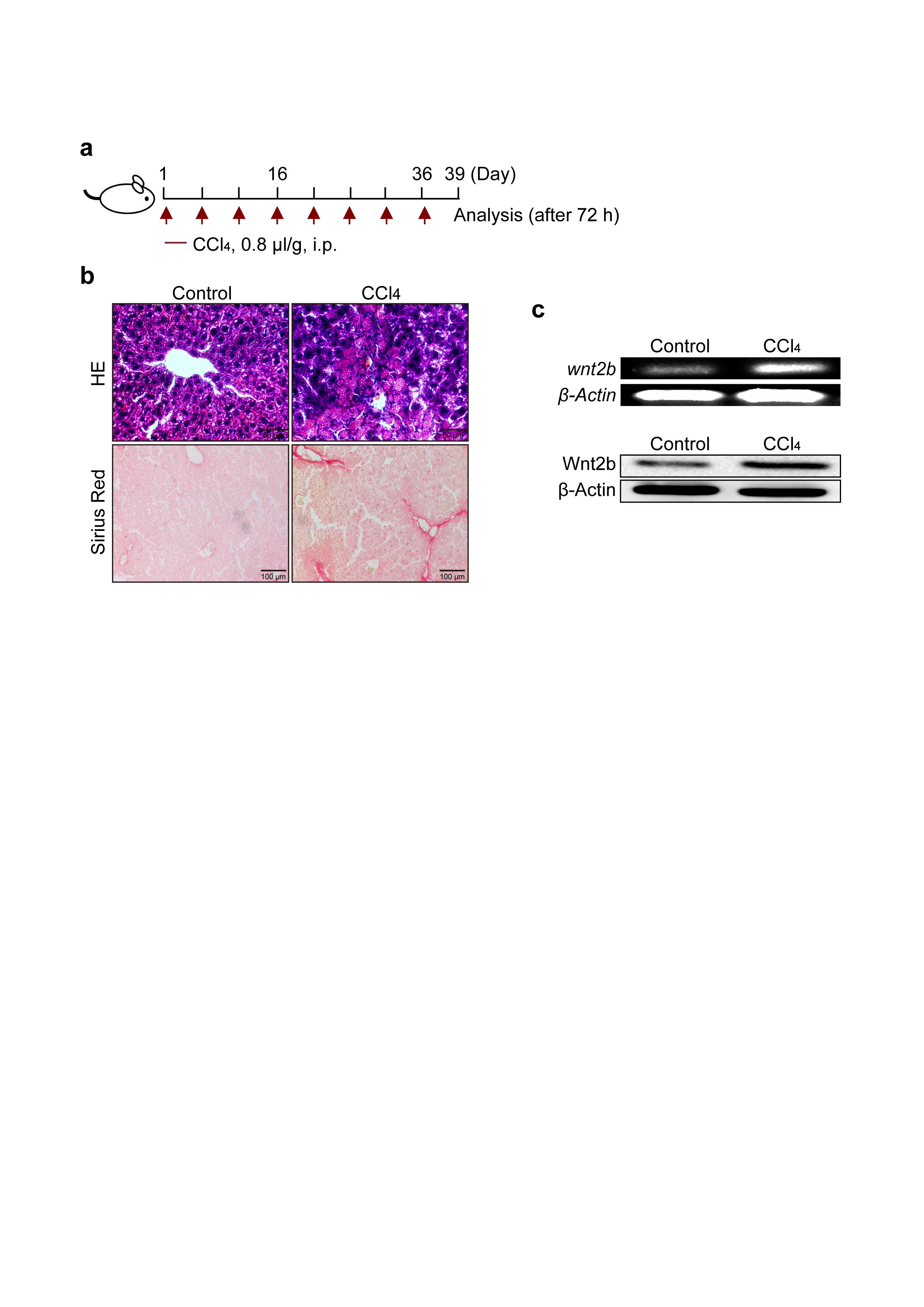

Supplementary Figure 1.** **Wnt2b expression is elevated in CCl_4_-induced hepatic fibrosis mouse model.** (**a**) Hepatic fibrosis mouse model was induced by 8 intraperitoneal injections of CCl_4_ at 0.8 ml/kg. Then the following analyses were performed. (**b**) H&E and Sirius Red staining, (**c**) RT-PCR (upper) and Western blot (lower) analysis of Wnt2b of liver tissues. Statistical analyses provided the mean ± SE (n = 3/group), * *P* <0.05, ** *P* <0.01, *** *P* <0.001.


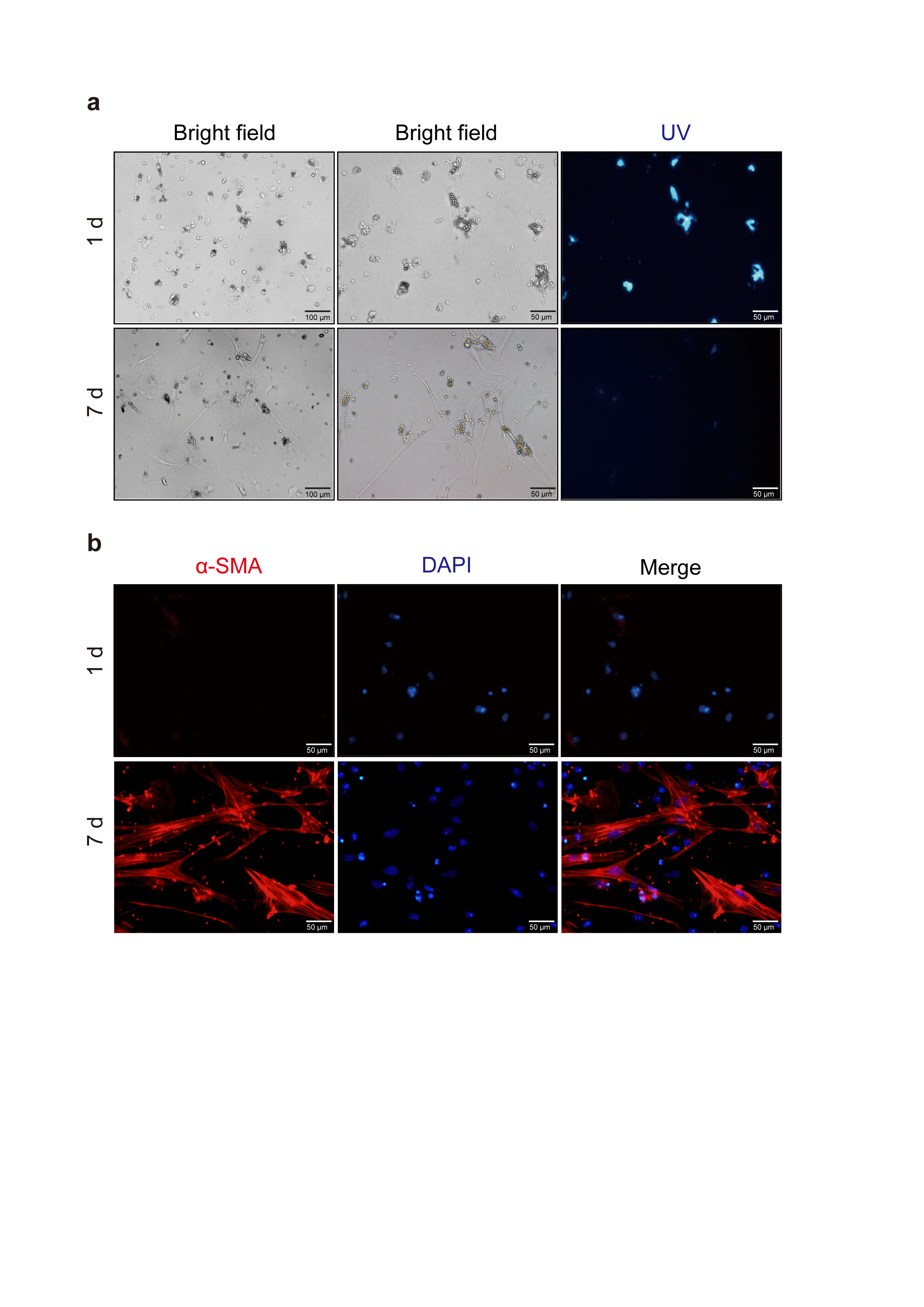

**Supplementary Figure 2.** **Characterization of hepatic stellate cells.** HSCs were isolated from naive mouse livers as described in Materials and Methods. The images depicted HSCs on day 1 (quiescent phenotype) and on day 7 (activated phenotype) of *in vitro* culture. (**a**) Quiescent HSCs exhibited high amounts of lipid droplets, which excited auto-fluorescence under ultraviolet light (upper), while activated HSCs gradually lost auto-fluorescence and acquired distinctive lipid vesicles morphology (lower). (**b**) Compared with quiescent phenotype (upper), activated HSCs (lower) expressed more α-SMA (shown in red) as demonstrated by immunofluorescence staining. Data are representative of at least three different experiments.

**
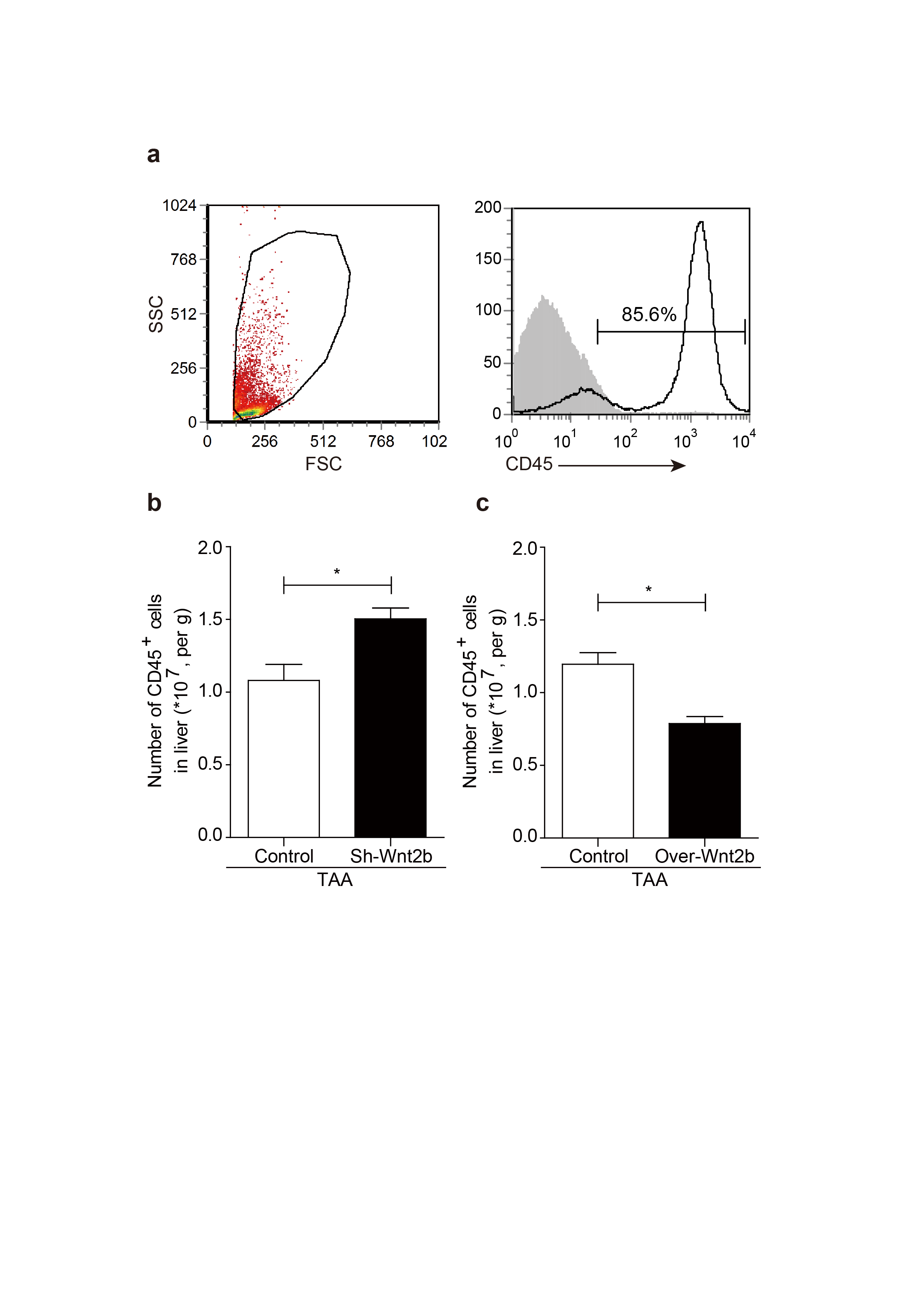
**

**Supplementary Figure 3. Flow Cytometry Analysis and Quantification of CD45 positive cells within fibrotic livers.** The extent of immune cell infiltration was quantified by analyzing the number of hepatic CD45 positive cells from mice challenged with TAA combined with HD injection of sh-Wnt2b / pRK5-mWnt2b construct or control vector as depicted in Figure 3. (**a**) The purity of isolated CD45 positive cells was confirmed by flow cytometry. (**b, c**) The absolute number of hepatic CD45 positive cells (per g of the liver) in Wnt2b-silenced (**b**)/Wnt2b-overexpressed (**c**) mice was quantified. Statistical analysis provided the mean ± SE (n = 3/group), * *P* <0.05, ** *P* <0.01, *** *P* <0.001.

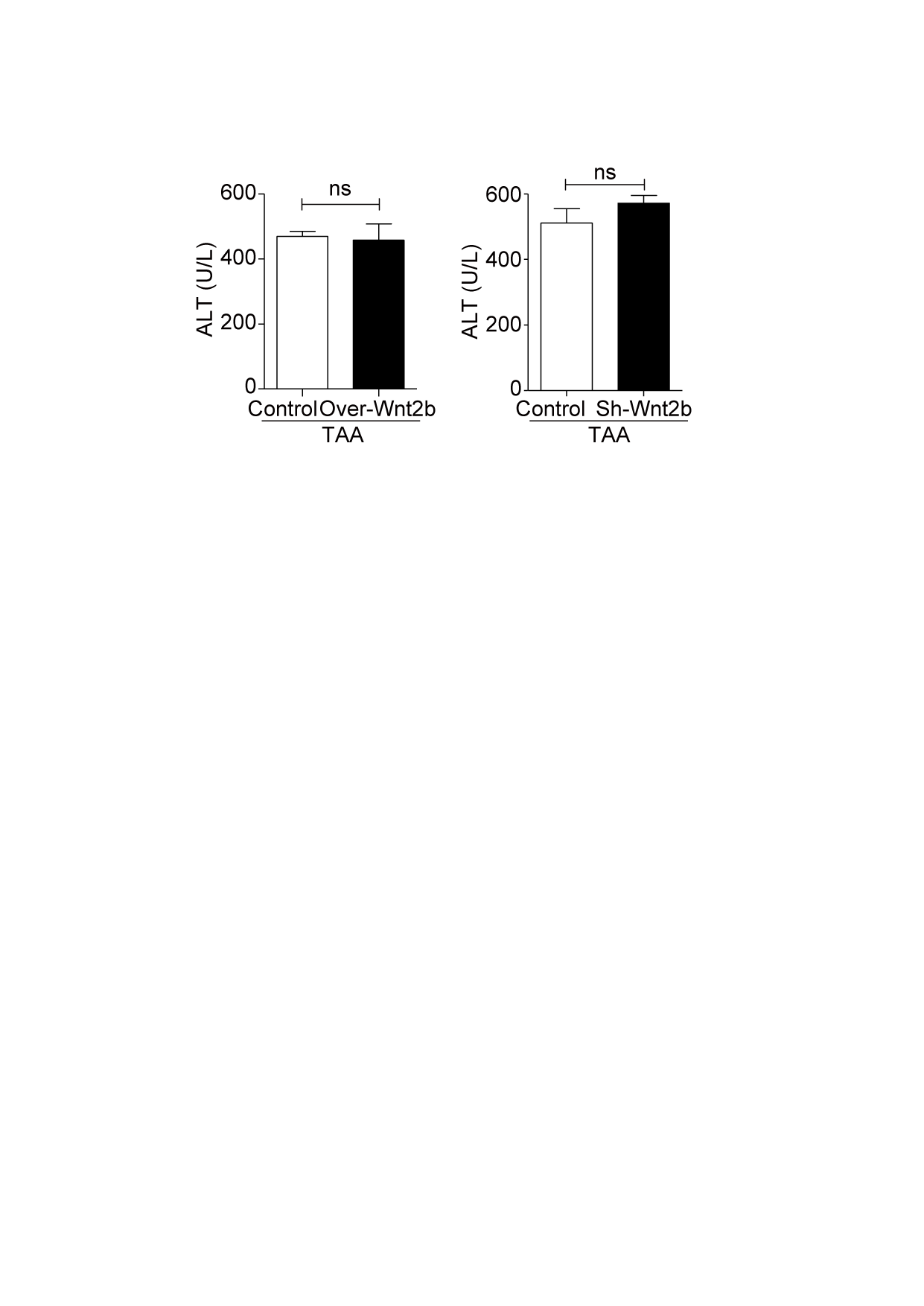

**Supplementary Figure 4.** **Analysis of the serum ALT levels.** Serum was collected as described in Fig. 3a. Compared to that in control mice, ALT levels were not significantly changed in Wnt2b-overexpressed (left)/Wnt2b-silenced (right) mice. Statistical analyses provided the mean ± SE (n = 6/group), * *P* <0.05, ** *P* <0.01, *** *P* <0.001.


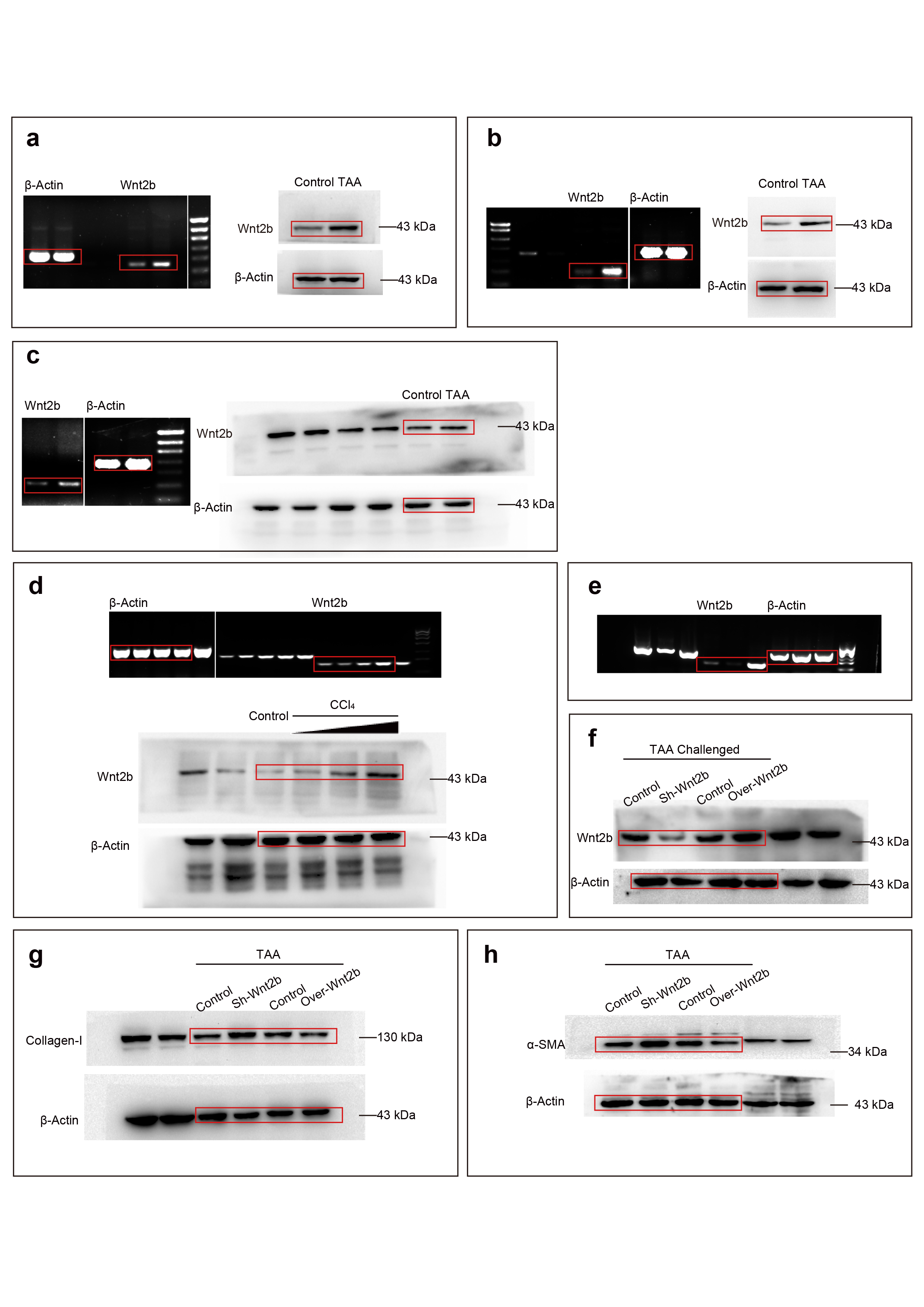
 **Supplementary Figure 5.** **The full-length blots are the display of cropped blots from the main Figure 1, 2, 3.** (**a**) For **Fig. 1d**. (**b, c**) For **Fig. 2a**. (**d**) For **Fig. 2b**. (**e**) For **Fig. 2d**. (**f**) For **Fig. 3b**. (**g**) For **Fig. 3c** and **Fig. 3e**. (**h**) For **Fig. 3d** and **Fig. 3f**. The samples for detecting Wnt2b and β-Actin were derived from the same experiment and that blots were processed in parallel.


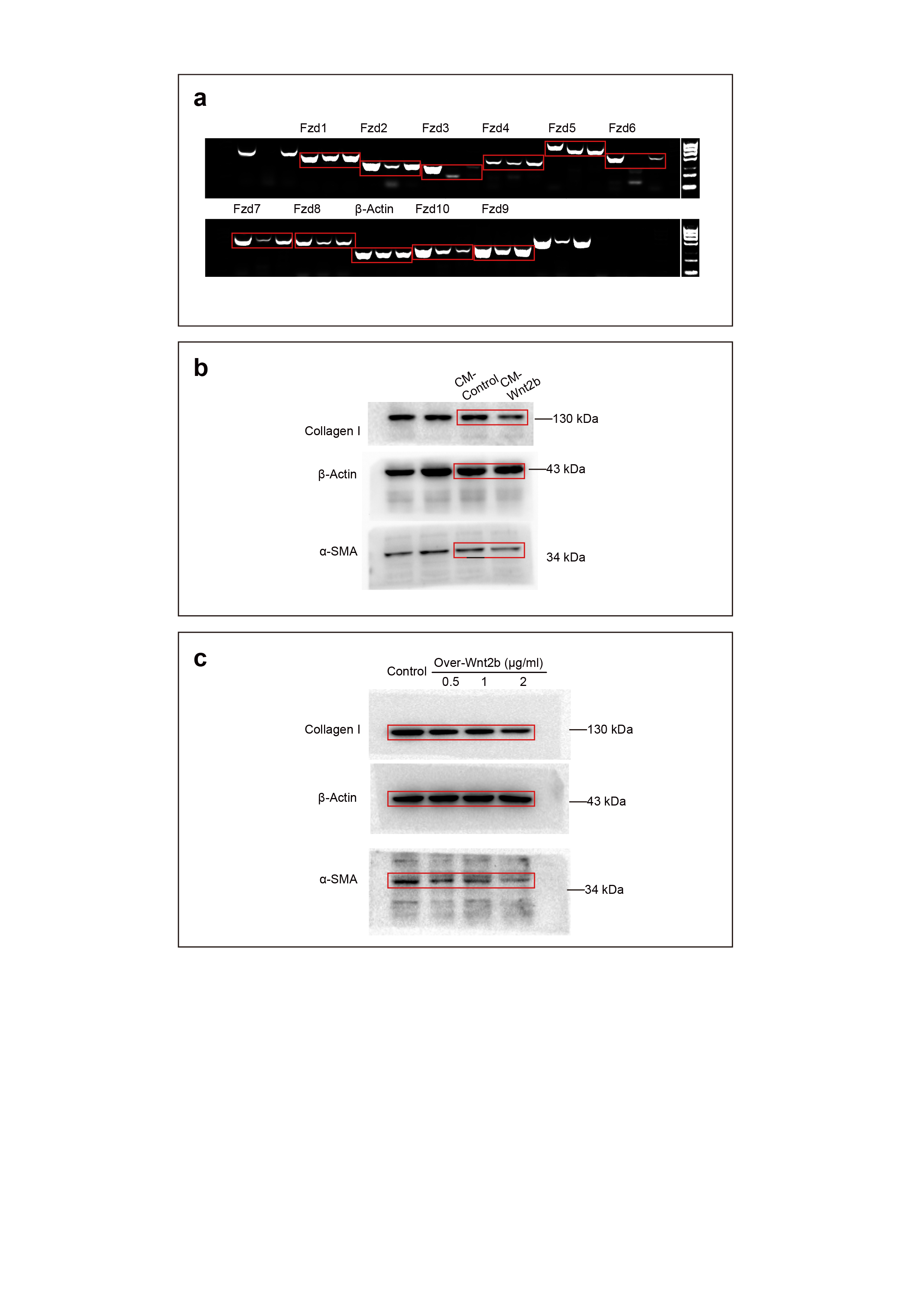


**Supplementary Figure 6.** **The full-length blots are the display of cropped blots from the main Figure 4.** (**a**) For **Fig. 4a**. (**b**) For **Fig. 4b**. (**c**) For **Fig. 4c**.


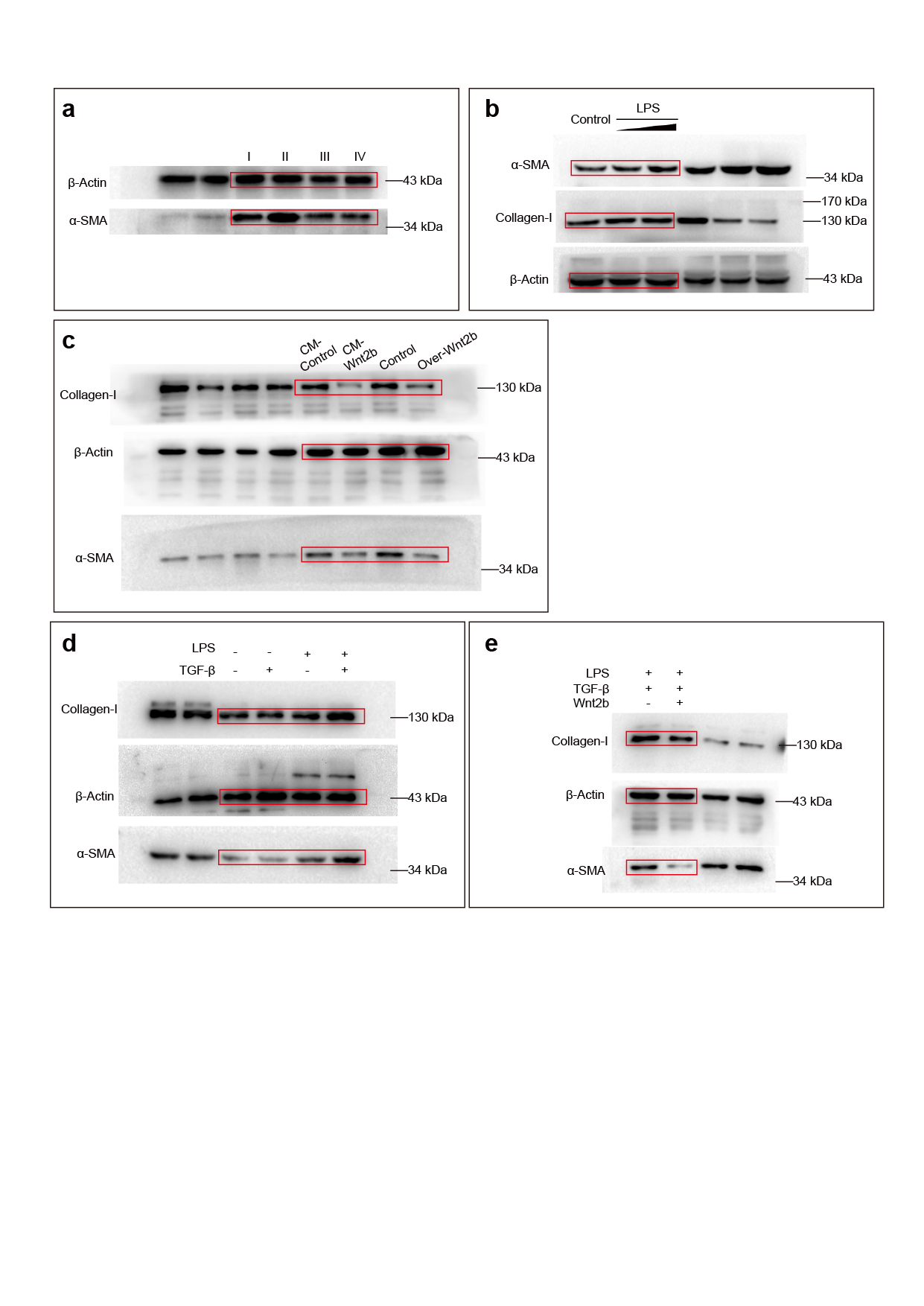


**Supplementary Figure 7.** **The full-length blots are the display of cropped blots from the main Figure 5.** (**a**) For **Fig. 5c**. (**b**) For **Fig. 5d**. (**c**) For **Fig. 5e**. (**d**) For **Fig. 5f**. (**f**) For **Fig. 3b**. (**e**) For **Fig. 5g**.


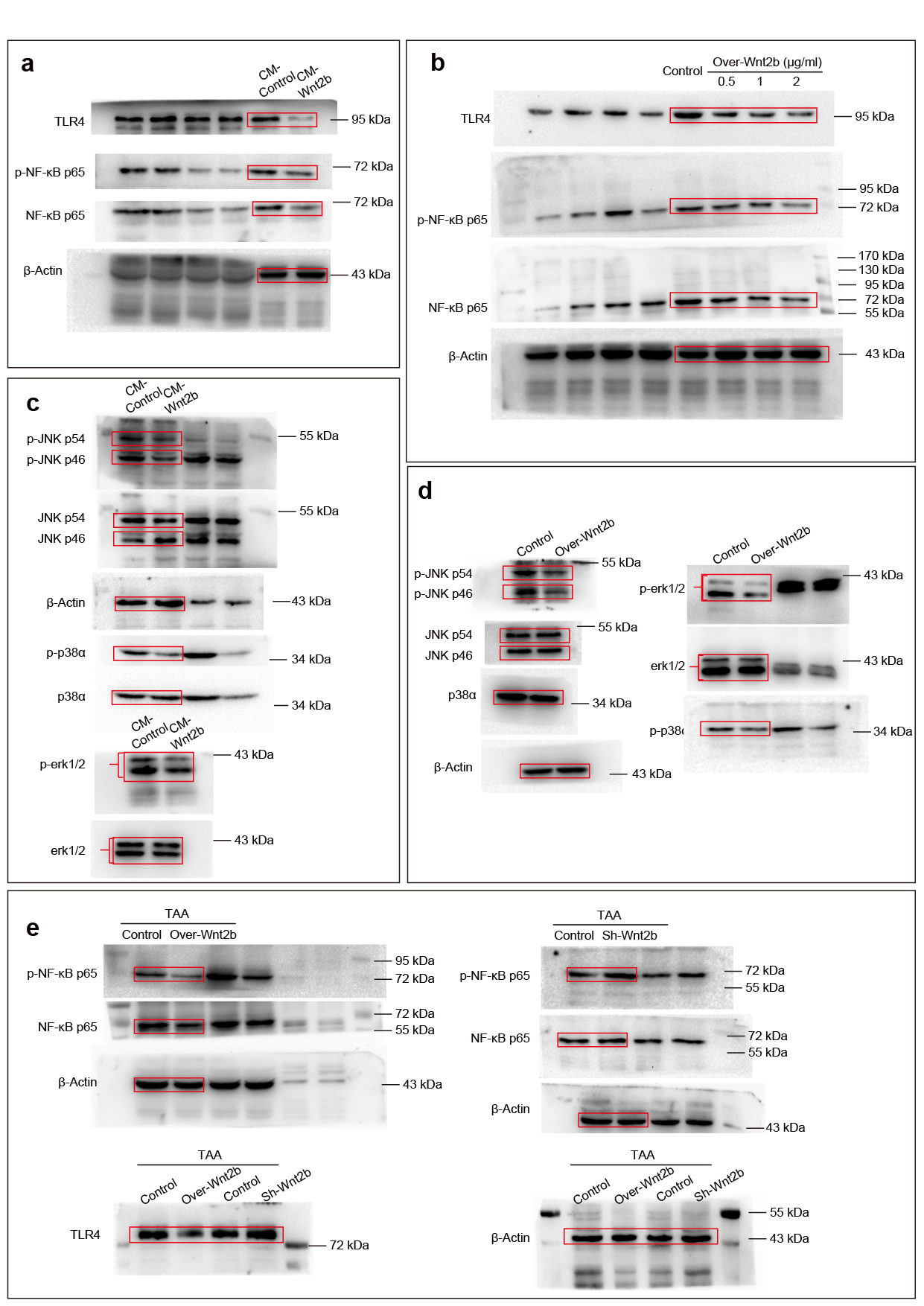


**Supplementary Figure 8.** **The full-length blots are the display of cropped blots from the main Figure 6.** (**a**) For **Fig. 6a**. (**b**) For **Fig. 6b**. (**c**) For **Fig. 6c**. (**d**) For **Fig. 6d**. (**e**) For **Fig. 6e**. The samples for detecting MAPKs and β-Actin were derived from the same experiment and that blots were processed in parallel.
